# Advances in T-Cell Epitope Engineering

**DOI:** 10.3389/fimmu.2013.00133

**Published:** 2013-06-05

**Authors:** Johanne M. Pentier, Andrew K. Sewell, John J. Miles

**Affiliations:** ^1^Institute of Infection and Immunity, Cardiff University School of MedicineHeath Park, Cardiff, Wales, UK; ^2^Human Immunity Laboratory, Queensland Institute of Medical ResearchBrisbane, QLD, Australia; ^3^School of Medicine, The University of QueenslandBrisbane, QLD, Australia

## Introduction

T-cells recognize small peptide fragments (p) cradled in multiple major histocompatibility complex (MHC) molecules, termed *human leukocyte antigens* (*HLA*) in humans. These membrane-integral pMHC molecules are present on surface of all nucleated cells and allow T-cells to detect aberrant intracellular activity, be this infection with microorganisms or abnormal host biochemistry such as neoplastic division. Scanning of pMHC molecules occurs via the αβ T-cell receptor (TCR), a clonotypic, heterodimeric, and membrane-integral molecule on the T-cell surface (Miles et al., 2011a). TCRs engage pMHC molecules via six highly flexible complementarity determining region (CDR) loops and, upon productive docking with a pMHC molecule, the TCR triggers a myriad of intracellular T-cell signaling cascades (Bridgeman et al., 2012). The binding strength (or affinity) between a TCR and a cognate pMHC is relatively weak across known biological systems with monomeric “dwell times” (or half-lives) typically measured in seconds or microseconds at physiological temperatures (Miles et al., 2010; Bridgeman et al., 2012; Smith et al., 2012). This is in contrast to numerous other biological interactions such as antibodies (van der Merwe and Davis, 2003), interleukins (Morton et al., 1994), lipoproteins (Misra et al., 2001), and structural membrane proteins (Matte et al., 2012) which have half-lives measured in hours-to-days. Overall, TCR/pMHC interactions are fleeting even by the dynamic standards of cell surface interactions (van der Merwe and Davis, 2003). The evolutionary rationale for this striking functional divide can only be speculated upon but likely pertains to the primary function of T-cells. T-cells must scan large numbers of pMHC on multiple cells in series in order to identify and eliminate threats quickly. Effective immunity requires that TCR scanning time must be minimal and antigen coverage maximal. Theoretical arguments dictate that maximal immune cover of possible foreign pMHC requires each TCR to recognize huge numbers of different peptides (Mason, 1998; Sewell, 2012). This theory is now supported up by direct experimental evidence that shows a single TCR can cross-recognize millions of pMHC molecules as well or better than the native antigen (Sewell, 2012; Wooldridge et al., 2012; Ekeruche-Makinde et al., 2013). Curiously, this extensive T-cell cross-reactivity is strictly compartmentalized based on peptide length (Ekeruche-Makinde et al., 2013).

An interesting consequence of the low antigen affinity and high antigen cross-reactivity characteristics of TCRs is that many, and perhaps all, cognate antigens could potentially be improved upon. Through rational structural modifications of native blueprint antigens we now know it is possible to engineer “optimal fit antigens” which exhibit logarithmic increases in affinity and immunogenicity. Compared with the native antigens, if these optimal antigens prove more effective at stimulating antigen-specific T-cell populations during experimental priming then the compounds may fundamentally redefine how we think about vaccine design.

## Improving T-Cell Epitopes

T-cell epitopes can be optimized by: (i) improving antigen affinity for MHC; (ii) improving antigen affinity for TCR; and (iii) improving antigen pharmacology through synthetic biology. Enhancing the stability of the epitope within the MHC cleft is the simplest engineering strategy as MHC anchoring preferences have been determined from MS elution data (Rammensee et al., 1995) resulting in MHC-binding databases and MHC-binding prediction algorithms (Rammensee et al., 1999; Wang et al., 2011). In theory, the replacement of buried suboptimal anchor residues with optimal residues for MHC-binding will produce a more stable pMHC complex and improved recognition of peptide. Recent studies have shown that things are not straightforward and this simple strategy for antigen improvement requires careful evaluation. We now know that anchor residue-modified peptides can have minimal or no improvement on stability in the MHC cleft (Miles et al., 2011b). Additionally, previous work has shown that MHC-binding strength shows little correlation with immunogenicity (Assarsson et al., 2007). We have recently shown that anchor residue-modified peptides can substantially alter TCR binding in ways that are difficult to predict and thereby prime T-cells with altered TCR repertoires (Ekeruche-Makinde et al., 2012). These repertoire effects have clinical relevance as it was found that vaccination with anchor residue-modified peptides was less effective than vaccination with natural peptides at priming tumor-specific T-cells in patients (Speiser et al., 2008).

T-cell epitopes can also be improved by optimizing contact interface between the peptide and TCR. In its simplest form this can be achieved by scanning recognition of a monosubstituted analog library (MAL) (Burrows et al., 1992, 1995; Zaremba et al., 1997; Tangri et al., 2001; Kjer-Nielsen et al., 2003; Burrows, 2004; Bulek et al., 2012). MALs substitute one of all available proteogenic amino acids across each position of a peptide backbone. While this approach can rapidly identify optimal antigens it is expensive as it requires the manufacture a unique defined analog library for each epitope examined. Combinatorial peptide libraries (CPLs) provide more flexible approach for the identification of optimal ligands (Borras et al., 2002). These very large, mixture-based compound libraries are synthesized in positional scanning format so that just one position along the peptide backbone is a fixed amino acid and all other positions are degenerate, with degenerate positions containing any one of 19 proteogenic amino acids (cysteine is excluded to reduce disulfide bond formation within the compound mixtures). Scanning a CPL across a T-cell clone can quantitatively map which residue/s are preferred by the TCR along a peptide backbone even if true antigen specificity of the clone is unknown. Replacing native residues with preferred residues identified by CPL can significantly increase epitope affinity and immunogenicity (see table below). The advantage of CPL scanning is that the same compound library can be used for any T-cell from any system although it important to use a library of correct peptide length (Ekeruche-Makinde et al., 2012).

An alternative strategy for T-cell epitope optimization focuses on improving compound pharmacology by artificially engineering a compound to mimic a peptide (a peptidomimetic). The motive behind this strategy is that proteogenic amino acid-based polypeptides are susceptible to acid degradation and rapid proteolytic cleavage and have half-lives of less than 5 min in the presence of proteases (Guichard et al., 1994) or human serum (Stemmer et al., 1999). Replacing proteogenic amino acids with synthetic subunits generates resilience to proteases and has potential to vastly improve compound shelf life and *in vivo* bioavailability during prophylactic and therapeutic applications. Many synthetic subunits can be substituted for proteogenic amino acids to impede proteolysis. These include D-amino acids (Bartnes et al., 1997), β-amino acids (Webb et al., 2005), psi-bonded amino acids (Stemmer et al., 1999), and the shifting of the R group by one atom to create poly-*N*-substituted glycines (peptoids) where the side chains are appended to the nitrogen atom of the peptide backbone (Gocke et al., 2009).

## Why Optimize T-Cell Epitopes?

We know that the *number* of antigen-specific T-cells within the host has fundamental relevance for disease control. For example the number of virus-specific T-cells generated during primary infection shows an inverse correlation with viral load (Ogg et al., 1998; Bharadwaj et al., 2001) and, in multiple cancer vaccine trials, the number of tumor-specific T-cells circulating within a patient and at tumor site/s correlates with clinical response (Lonchay et al., 2004; Rosenberg et al., 2004). While T-cell numbers alone do not determine disease outcome *per se*, global numbers are central in tipping control toward the host. With this in mind, engineering potent new compounds aimed at amplifying defined subsections of cellular immunity is the first step in manipulating the T-cell compartment for the purposes of rational vaccine design and therapeutic intervention. Such endeavors are particular important for cancer (self) antigens which generally exhibit very low immunogenicity (Chatten and Bathe, 2010).

## Examples of T-Cell Epitope Optimization

Table 1 presents examples of MHC-anchor based (Parkhurst et al., 1996; Bernatchez et al., 2011), MAL-based (Burrows et al., 1992; Zaremba et al., 1997; Tangri et al., 2001; Terasawa et al., 2002), CPL-based (La Rosa et al., 2001; Blondelle et al., 2008; Ekeruche-Makinde et al., 2012; Wooldridge et al., 2012), and synthetic ligand-based (Mézière et al., 1997; Stemmer et al., 1999; Webb et al., 2005) T-cell epitope optimization across mice and humans. Collectively, this work demonstrates that T-cell epitopes can be readily optimized across both foreign and self peptide “blueprints” to increase sensitivity to T-cells and/or increase compound stability. Both MAL-based and CPL-based platforms appear equally effective at generating optimized ligands and have been used to increase antigen sensitivity between 100- and 10,000-fold relative to the native epitope blueprint. It is of particular interest to note that it is not just self self-derived epitopes that can have their immunogenicities amplified logarithmically. One CPL study (La Rosa et al., 2001) generated optimal ligands based on the HLA-A^*^-0201-binding epitope NLVPMVATV (A2-NLV) blueprint from human cytomegalovirus (HCMV) which were 10,000-fold more active. This achievement is striking given that the native A2-NLV epitope is one of the most immunogenic across the HCMV proteome (Khan et al., 2004). Thus, even very good T-cell antigens can be improved upon by modification in amino acid sequence. There is now accumulating evidence demonstrating that T-cells recognize a variety of molecular shapes presented by self MHC molecules (Bridgeman et al., 2012). These molecular shapes need not be made from L-amino acids as demonstrated by recognition of peptidomimetics. To date, such compounds have been made by rational design and have failed to exceed the potency of optimal peptide ligands. It is possible that the screening of combinatorial chemistries so that T-cells can select their recognition preference may overcome this limitation. While these synthetic compounds are unable, at this time, to exceed the sensitivity of the model epitope during completive titrations, they are considerably more resistant to digestion from proteases and serum and demonstrate remarkable biostability.

**Table 1:** **Examples of T-cell epitope optimization**.

Species	Disease	Model Ag	Epitope	MHC	Modification	Functional improvement	Reference
Human	EBV	EBNA 3A	FLRGRAYGL	B*0801	MAL-directed substitution	100 fold increase in sensitivity	Burrows et al. (1992)
Human	CMV	pp65	NLVPMVATV	A*0201	CPL-directed substitution	1,000 to 10,000-fold increase in sensitivity	La Rosa et al. (2001)
Mouse	N/A	Ovalbumin	SIINFEKL	H-2Kb	β-amino acid insertion	500% more stable during serum digestion	Webb et al. (2005)
Mouse	LCMV	glycoprotein	KAVYNFATM	H-2Db	Psi bond insertion	20-fold more stable during protease digestion	Stemmer et al. (1999)
Mouse	keratitis	IgG2a	YFMYSKLRVQKSC	I-Ad	D-amino acid retro-inverso	As active in vivo as the proteogenic peptide	Mézière et al. (1997)
Human	cancer	MAGE/CEA	various	A*0201	MAL-directed substitution	Up to 10,000-fold increase in sensitivity	Tangri et al. (2001)
Human	cancer	gp100	various	A*0201	MHC-anchor substitution	More numbers of T-cells recovered after in vitro prime	Parkhurst et al. (1996)
Human	cancer	PSA	VISNDVCAQV	A*0201	MAL-directed substitution	Better able to induce T-cell activation	Terasawa et al. (2002)
Human	cancer	CEA	YLSGANLNL	A*0201	MAL-directed substitution	1,000-fold increase in sensitivity	Zaremba et al. (1997)
Human	HIV	Gag	TLNAWVKVV	A*0201	CPL-directed substitution	130% increase of T-cells recovered after in vitro prime	Blondelle et al. (2008)
Human	cancer	survivin	ELMLGEFLKL	A*0201	MHC-anchor substitution	Induces stronger T-cell responses in 30% of donors	Bernatchez et al. (2011)
Human	cancer	Melan A	AAGIGILTV	A*0201	CPL-directed substitution	500% increase in TCR/pMHC-binding affinity	Ekeruche-Makinde et al. (2012)
Human	diabetes	preproinsulin	ALWGPDPAAA	A*0201	CPL-directed substitution	100 to 1,000-fold increase in sensitivity	Ekeruche-Makinde et al. (2013)

*MAL, monosubstituted analog library; CPL, combinatorial peptide library*.

## Where Next?

It is clear that optimal T-cell epitopes can be generated with relative ease but the big question of whether they really work as therapeutic and prophylactic vaccines remains unanswered. Future studies should determine whether optimal T-cell epitopes can outperform native ligands during *in vitro* priming from healthy and patient samples. We also need to determine what effects priming with optimal T-cell epitopes has on the responding T-cell repertoire, both in terms of polyfunctionality and TCR usage. Studies that directly test whether optimal T-cell epitopes can outperform native ligands during *in vivo* priming of naïve animals are required. Importantly, these responses must be tested in relevant *in vivo* models of infection. It is hoped that optimal epitopes can be found that amplify greater numbers of T-cells than the native ligand alone and that T-cells maintain the desired functional profiles required for effective immunity. The development of effective synthetic T-cell epitopes will be particularly exciting as these epitopes have many attractive characteristics. First, they can be engineered to show absolute resistance to temperature fluctuations and environmental degradation, effectively nullifying the need for cold chain storage and transport. Indeed, the cold chain transport of vaccines costs WHO–UNICEF around USD 11.5 billion every year (Wolfson et al., 2008). Second, the acid- and protease-resistant properties of synthetic compounds raise the possibility of orally active administration. Here, the needle-free nature of compound release would considerably decrease logistical problems with vaccine service delivery, significantly decrease systems costs and is likely to increase vaccine acceptance rates. T-cell sensitivity toward the first generation of “designed” synthetic epitopes has been slightly weaker when compared with proteogenic epitopes. However, we believe that that there is ample scope to improve upon proteogenic epitopes by screening the right sorts of combinatorial chemistry. Our recent preliminary data with such a synthetic epitope shows that it can produce strong responses when given orally and that the T-cells induced against this compound protect humanized mice against a lethal challenge with influenza. Further studies are required in order to see whether such an approach can be extended to other T-cell epitopes.

In summary, optimization of T-cell epitopes can be achieved using a number of different techniques. Whether the resultant compounds can be used as effective prophylactic or therapeutic vaccines in humans remains to be determined. However, such approaches may allow the precise targeting of the most effective T-cell clonotypes (Ekeruche-Makinde et al., 2012) *in vivo* and could have the potential to change the way we build vaccines and immunotherapies in the future.
